# Relationships Between Ion Channels, Mitochondrial Functions and Inflammation in Human Aging

**DOI:** 10.3389/fphys.2019.00158

**Published:** 2019-03-01

**Authors:** Marie Strickland, Besma Yacoubi-Loueslati, Balkiss Bouhaouala-Zahar, Sylvia L. F. Pender, Anis Larbi

**Affiliations:** ^1^Singapore Immunology Network, Agency for Science Technology and Research, Singapore, Singapore; ^2^Clinical and Experimental Sciences, Faculty of Medicine, University of Southampton, Southampton, United Kingdom; ^3^Laboratory of Mycology, Pathologies and Biomarkers, Department of Biology, Faculty of Sciences, University Tunis El Manar, Tunis, Tunisia; ^4^Laboratory of Venoms and Therapeutic Molecules, Institut Pasteur de Tunis, University Tunis El Manar, Tunis, Tunisia; ^5^Medical School of Tunis, University Tunis El Manar, Tunis, Tunisia; ^6^Chinese University of Hong Kong – University of Southampton Joint Lab for Stem Cell and Regenerative Medicine, Hong Kong, China; ^7^Department of Biology, Faculty of Sciences, University Tunis El Manar, Tunis, Tunisia; ^8^Department of Microbiology and Immunology, Yong Loo Lin School of Medicine, National University of Singapore, Singapore, Singapore; ^9^Geriatrics Division, Department of Medicine, Research Center on Aging, University of Sherbrooke, Sherbrooke, QC, Canada

**Keywords:** aging, mitochondria, ion channels, inflammation, cellular senescence

## Abstract

Aging is often associated with a loss of function. We believe aging to be more an adaptation to the various, and often continuous, stressors encountered during life in order to maintain overall functionality of the systems. The maladaptation of a system during aging may increase the susceptibility to diseases. There are basic cellular functions that may influence and/or are influenced by aging. Mitochondrial function is amongst these. Their presence in almost all cell types makes of these valuable targets for interventions to slow down or even reserve signs of aging. In this review, the role of mitochondria and essential physiological regulators of mitochondria and cellular functions, ion channels, will be discussed in the context of human aging. The origins of inflamm-aging, associated with poor clinical outcomes, will be linked to mitochondria and ion channel biology.

## Aging: A Physiological Adaptation

### Chronological Aging

The definition of aging has been of great debate between scientists. Many view aging as a disease itself, whilst others describe aging as the inevitable decline of function with time which increases propensity to age-related disease development. Victor Hugo said: “*Forty is the old age of youth, fifty is the youth of old age*” already suggesting strata to exist within each of the periods of life. In this review, we define aging as individuals in the last stage of their life, often represented in the literature as retirement age (65 years old). The life expectancy in developed countries is longer (83 years in Singapore, 81 years in United Kingdom). Nevertheless, most countries will be approaching a critical demography during this century, including extended life expectancy (75 years in Tunisia), reduced birth rate and reduced workforce (World Health Organization, 2015). While aging of the population is currently seen as a socio-economic burden it is possible to make it an opportunity. The current view is highly influenced by the fact that many of the elderly also display diseases and disabilities (World Health Organization, 2015; Vos et al., 2017). For example, the decline of pulmonary, cardiovascular and immune systems has been shown in longitudinal aging studies, alongside physical decline such as sarcopenia and frailty (Ng et al., 2015; Shimokata and Ando, 2017; Huang et al., 2018). These may lead to loss of physical integrity, impaired function and vulnerability to death. Physiological aging can take two forms: (1) the normal decline in function, which occurs in all individuals with age, and (2) the loss of function from one or more diseases encountered with age (Hayflick, 1994). Whilst the first may be seen by many as untreatable, the second form often is treatable due to the great strides we have made in treating and controlling age-related diseases such as diabetes and atherosclerosis. In order to better integrate the elderly population in our societies it is of utmost importance to understand how to increase healthspan. While assistive technologies are necessary to fill the gap, the long-term challenge is to prevent the elderly from physical decline.

### Biological Aging

We view aging as an adaptation to lifelong events, and interventions should support the physiological balance during age-related adaptation, response to acute stress, in order to avoid disease onset. Adapted capacity in most organs has been shown to occur from the third and fourth decades of life (Boss and Seegmiller, 1981; Khan et al., 2017). Chronological age by itself is not a great predictor of aging, healthspan or functional status (Yang and Lee, 2009), with individuals of the same age putting vastly different demands on health care systems and society in general. Whilst the incidence of the top five chronic diseases increases with age, 32% of the participants aged 85 and over in the 2004 US Health and Retirement study were not diagnosed with these conditions. Additionally, 28% of those 85 and over reported themselves as in excellent or very good health compared to 48% of the 51–54 age bracket (Lowsky et al., 2014). The future of aging research may depend on our ability to stratify elderly populations and predict clinical trajectories of the pre-symptomatic adult populations. Young individuals of the same chronological age (38 years) have been found to vary in their biological age, measured by the functional decline of multiple organ systems prior to the onset of age-related disease (Belsky et al., 2015). Young individuals with an advanced pace of aging show increased physical and neurological decline compared to their slower aging counterparts between the ages of 26 and 38 (Belsky et al., 2015). This pool with a faster pace of aging could be used to evaluate the effectiveness of anti-aging therapies prior to disease onset.

Methods to assess biological age and the rate of aging are varied. Measurements can range from simple one-off measurements of telomere length to complex measurements of epigenetic ticking rate and algorithms of biomarkers taken over time (Belsky et al., 2018). Whilst agreement between different measures of biological aging is low, the 71-cytosine-phosphate-guanine epigenetic clock and biomarker algorithms were recently found to be more reliable than other methods in relation to physiological decline of organs and facial aging (Belsky et al., 2018). Recent focus has been on understanding more about epigenetic markers of aging (Mitnitski, 2018). Methylation within certain CpG sites correlates with age, and have formed the basis of epigenetic clock models of biological aging (Hannum et al., 2013). In humans, methylation states of specific genes appear to correlate well with biological age and link obesity and human immunodeficiency virus (HIV)-infection as accelerators of methylation and biological aging (Horvath et al., 2014; Gross et al., 2016). In fact, a 5-year increase in biological age as measured by epigenetic methylation results in a 21% increase in mortality risk (Marioni et al., 2015). Life span interventions such as calorie restriction and rapamycin, discussed later in this review, have previously been shown to reduce epigenetic age in mice, which show conserved CpG methylation sites (Wang et al., 2017).

### Core Physiological Functions in Aging

#### Cardiovascular

Cardiac output decreases by a rate of approximately 1% per year from the first decade of life, independently of cardiac disease. This is thought to occur through the senescence of cardiac muscle, decreased response of cardiac cells to glycosides and increased amyloid deposits with age (Steenman and Lande, 2017). These deposits also increase the prevalence of atherosclerosis and coronary artery disease in the elderly, severely affecting the cardiovascular system (Hansson, 2005). Alongside the respiratory and genito-urinary systems show reduced function with age as well as decreased immune function, leading to increased infection rates in the elderly (Kline and Bowdish, 2016; Nicolle, 2016; Chason et al., 2018). This suggests that several compartments of the immune system, including mucosal immunity, are altered in old age (Sala-Rabanal et al., 2015; Martelli et al., 2016).

#### Central Nervous System

Aging is associated with the development of numerous neuropathologies which appear exponentially with advancing age (Niccoli and Partridge, 2012). The accumulation of plaques in the brain has been associated with cognitive impairment and the development of Alzheimer’s disease, whilst mitochondrial dysfunction is a causative factor in the appearance of Parkinson’s disease (Pickrell and Youle, 2015; Snyder et al., 2015). In fact, the brain has been highlighted as particularly susceptible to aging. Dementia, an umbrella term for numerous neurodegenerative conditions, is a global issue affecting those from both low-income and high-income countries, the prevalence of which is expected to double every 20 years going forward (Prince et al., 2013). Numerous causes have been attributed to the development of neurodegenerative disorders, such as dysregulated calcium signaling impacting on neuronal signaling, altered mitochondrial and ion channel dynamics affecting mid-brain cell survival in Parkinson’s diseases and the contribution of “inflamm-aging” to neuroinflammation and decreased neuroplasticity (Di Benedetto et al., 2017; Frazier et al., 2017; Peng et al., 2018). Mitochondrial defects in particular have a large impact on neurological systems due to larger energetic requirements of the brain compared to other systems (Grimm and Eckert, 2017).

#### Metabolic

Progressive deterioration of the function of pancreatic beta cells and the development of insulin insensitivity causes one of the most prevalent age-related diseases, type 2 diabetes (Shaw et al., 2010; Marselli et al., 2014). Beta cells show reduced capacity to respond to glucose levels, resulting in reduced insulin output, reduced ability to control blood glucose levels and increased adiposity (Kahn, 2003). With adipocyte enlargement also comes a relative decrease in lean body mass due to muscle wastage (Kalinkovich and Livshits, 2017). This loss of muscle (sarcopenia) is due to the atrophy of muscle cells, generally as a consequence of a sedentary lifestyle. Sarcopenia together with frailty are two diseases that are highly associated with poor clinical outcomes such as increased fall incidence and hospitalization (Kramer et al., 2017). In parallel to muscle loss bone density is often reduced and hip-fracture is a classical condition of the elderly with poor balance and frailty (Kramer et al., 2017).

#### Immune System

Inflammation is a physiological process leading to the repair of tissues in response to exogenous and/or endogenous stress. However, it may establish a biological foundation of the pathophysiological process of frailty, since chronic inflammation generally induces detrimental consequences (Miller, 1996). Inflammation is frequently caused by an aged-related change in the immune system, known as immunosenescence. The chronic inflammation associated with immunosenescence is known as “inflamm-aging” which is accompanied by cytokine dysregulation. This phenomenon is marked by increased pro-inflammatory cytokine production and reduction in anti-inflammatory cytokines. The clinical consequences of this include increased risk of comorbidities such as bone, nutritional, and muscle metabolism disorders and mortality (Manolagas and Jilka, 1995; Cesari et al., 2004; Franceschi, 2007). Systemic inflammation was found to be implicated in the pathophysiology of neurodegenerative and cardiovascular disorders (Akiyama et al., 2000; Cesari et al., 2003; Lopez-Candales et al., 2017).

Aging affects aspects of both the adaptive and immune compartments of the immune system. Understanding of age-related changes in the adaptive immune system far outweighs that of the innate immune system (Montgomery and Shaw, 2015), with reductions in naïve T cell pools and increases in memory pools with age observed for some time (Nikolich-Zugich, 2014). Generally T cell and B cell functions are reduced with age in many individuals, leading to reduced antibody production and T cell receptor (TCR) signaling defects in the elderly (Frasca et al., 2016; Le Page et al., 2018). Innate immune responses have also been shown to be dampened during ageing, alongside the development of a mature composition (Montgomery and Shaw, 2015). Additionally, there is a concurrent increase in the pro-inflammatory profile of the innate immune system leading to inflamm-aging (Bailey et al., 2018). The effects of aging on the major populations of immune cells varies greatly depending on cell type (Montgomery and Shaw, 2015). Monocytes isolated from elderly individuals display reduced cytokine production following toll-like receptor (TLR) activation with age and reduced interferon production, whilst dendritic cells show loss of antigen-cross presenting capacity (Chougnet et al., 2015; Metcalf et al., 2017; Molony et al., 2017). However, the innate immune system has also been shown to be activated at the basal level with aging (Molony et al., 2018). Reduction of the immune response to activation and increased basal activation of the adaptive and immune systems with age bring the concepts of immunosenescence and inflamm-aging together.

Not all systems become faulty with age in every individual, but most individuals will display one or more faulty system as they age. While loss or decreased function of organs may lead to mild symptoms and maintenance of autonomy and quality of life, failure of primary systems may have harsher consequences. The accumulation of senescent cells in various systems is thought to be the cause of their reduced functionality (Childs et al., 2015). Recent studies in genetically engineered mice suggest the specific removal of senescent cells (p16 expressing cells) as an efficient strategy to recover functions in older mice (Baker et al., 2011). One of these studies utilizes an INK-ATTAC transgene for inducible elimination of p16^INK4A^ expressing cells, a protein which is used as a biomarker for senescence and increases with ageing in both rodents and humans (Krishnamurthy et al., 2004). This transgene has been shown to reduce the onset of age-related disease in a progeroid mouse model when activated in a life-long and late-life manner, and reduced the functional decline of many organs (Baker et al., 2016; Bussian et al., 2018). Additionally, the senolytic drug ABT263, which selectively kills senescent cells in a B-cell lymphoma (BCL)-2/BCL-xL-dependent manner has shown promise reducing premature aging of the hematopoietic system in progeroid mice (Chang et al., 2016). This technique may also show promise in humans due to the fact that normal and induced-senescent human fibroblasts treated with a Forkhead box (FOX)O4 interfering peptide selectively targets senescent cells (Baar et al., 2017). This result has been confirmed *in vivo* in both normal aging and fast-aging mice which showed a reduction in the development of aging phenotypes compared to control treated mice (Baar et al., 2017). There is currently a series of efforts to test senolytics and aging-delaying drugs (Childs et al., 2018; Niedernhofer and Robbins, 2018). Limitations of these strategies (i) they are not targeted as they do not provide yet organ-specific removal of senescent cells (senolytics) and (ii) the aging-delaying drugs, including repurposed drugs (metformin, rapamycin) as well as novel ones (spermidine), have not yet been fully characterized for side-effects and aging-specific mechanisms of action (Aliper et al., 2017; Madeo et al., 2018).

In order to delay aging interventions may target its main hallmarks: genomic instability, telomere attrition, epigenetic alteration, loss of protein homeostasis, deregulated nutrient sensing, mitochondrial dysfunction, cellular senescence, stem cell exhaustion and altered intercellular communication (López-Otín et al., 2013). The unbalance in proteostasis has been suggested to be a significant player in the process of aging (Labbadia and Morimoto, 2015). Many upstream physiological systems such as ion channels function directly influence proteostasis as well as several other cellular processes (López-Otín et al., 2013; Hou et al., 2018). The same applies to mitochondria, an essential machinery linked to energy production and utilization in cells. The modulation of ion channel functions and mitochondrial activity are likely to have a wide range of effects depending on the cells/organs affected. The adaptation occurring during aging, and to some extent in senescent cells, has been described as a metabolic shift under intense mitochondrial influence (Wiley and Campisi, 2016). This further suggests aging and the accumulation of senescent cells to be driven by a metabolic shift and that modulation of the mitochondrial capacity may delay signs of aging. Hence, the control of essential systems such as ion channels and mitochondria would enable to reduce the pace of aging and promote healthspan. In this review, we focus on two aspects of cell physiology: ion channel biology and mitochondrial function that are interconnected and related to the majority of hallmarks of aging. Mitochondria have gained recent interest in the field of aging biology, especially since the discovery of autophagy and its role in proteostasis. Ion channel function is an overlooked phenomenon in the field of aging and this review aims to bring the attention to the topic.

## The Biology of Ion Channels in Aging

### Ion Channels Function and Dysfunction

Ion channels represent a variety of transmembrane proteins forming pores. These pores are selective to specific ions able to actively cross between intracellular and extracellular compartments, therefore mediating the influx and efflux of charged ions (Kulbacka et al., 2017). Their large structural diversity at monomeric and heteromeric levels, due to alternative splicing, supports their large functional diversity. Each cell type represents an assemblage of ion channels that shape the amplitude and duration of the action potential differently (Hoppa et al., 2014; Rowan et al., 2016). At the intracellular level, ion channels are also present on the surface of the mitochondria, endoplasmic reticulum and nuclear membrane (Charpentier et al., 2016; Raffaello et al., 2016).

Since the first structural resolution of Potassium (K^+^), Chloride (Cl^-^) and later on Sodium (Na^+^) channels by MacKinnon and Catterall research teams (Doyle et al., 1998; Dutzler et al., 2002; Payandeh et al., 2011), the biology of a large variety of ion channels has been well established. The development of a large set of biological small and active molecules targeting channels has been key to better understand their mechanisms of action and regulation (i.e., specific toxins, ligands, antibodies). About 400 annotated ion channel genes are retrieved in gene databases (about 1.5% of the human genome). Behind several structural similarities, their modes of action differ depending on the involved ion, the ion channel gating and permeation pathway (Yang and Nerbonne, 2016; Latorre et al., 2017). They are classified into various voltage-gated [Na^+^, K^+^, Cl^-^ and Calcium (Ca^2+^)] and ligand-gated ion channels [nicotinic acetylcholine receptors (nAChRs), γ-amino butyric acid (GABA), *N*-methyl-D-aspartate receptors (NMDARs), ryanodine receptors (RyRs)] according to their electrophysiological properties and their depolarization events, neuronal signaling and contraction in response to depolarization. According to their electrochemical gradients, Na^+^, K^+^, and Ca^2+^ channels and their respective ions cross the membrane when depolarization is activated (Bose et al., 2016). The electrophysiology reports of ion channel functions classify them into a large variety of subtypes according to their pharmacology and ionic properties, probably evolved from a common ancestor (Table 1). Despite this, chloride channels are distinctly classified as members of the voltage-sensitive subfamily with rarely predictable physiology [i.e., calcium-activated, high (maxi) conductance, cystic fibrosis transmembrane conductance regulator (CFTR) and volume regulated channels].

**Table 1:** Voltage-gated and ligand-gated ion channel nomenclature.

Gating classification	Channel family	Channel subunit/subfamily	Channel abbreviation
Voltage-gated ion	Voltage-gated sodium channel (Na^+^)	Alpha subunit	Na_V_1.1-1.9
channel		Beta subunit	Na_V_β1-4
	Voltage-gated calcium channel (Ca^2+^)	L-type calcium channel (Long-lasting)	Ca_V_1.1-1.4
		P/Q-type channel (Purkinje/Unknown)	Ca_V_2.1
		N-type channel (Neural)	Ca_V_2.2
		R-type channel (Residual)	Ca_V_2.3
		T-type channel (Transient)	Ca_V_3.1-3.3
	Voltage-gated potassium channel	Alpha subunit	K_V_1-12
		Beta subunit	K_V_β1-3
	Hyperpolarization-activated cyclic nucleotide-gated (CNG) channels	Alpha subunit	CNGα1-4
		Beta subunit	CNGβ1/3
	Voltage-gated proton channels	Hydrogen channel	H_V_1
Ligand-gated ion channel	Ionotropic glutamate receptors	α-amino -3-hydroxy-5-methyl-4-isoxazolepropionic acid receptor (AMPA)	GluA1-4
		Kainate	GluK1-5
		*N*-methyl -D-aspartate receptor (NMDA)	GluN1, NRL1A-B, GluN2A-D, GluN3A-B
		Orphan	GluD1-2
	Cys-loop receptors	Serotonin (5HT)	5-HT3A-E
		Nicotinamide acetylcholine (nAChR)	α1-10, β1-4, γ, δ, ε
		Zinc-activated ion channel (ZAC)	ZAC
		*Gamma-*aminobutyric acid (GABA)_A_	α1-6, β1-3, γ1-3, δ, ε, π, θ, ρ1-3
		Glycine (GlyR)	α1-4, β
	GABA receptors	GABA_A_ receptor	α1-6, β1-3, γ1-3, δ, ε, π, θ
	5-HT_3_ receptor	Serotonin	5-HT_3A-E_
	ATP-gated channels	ATP-gated P2X receptor	P2X_1-7_
	Phosphatidylinositol 4,5-biphosphate (PIP_2_)-gated channels	Inwardly rectifying potassium channel (K_ir_)-activating	K_ir_1.1-7.1


Malfunctioning and/or overexpression of ion channels has been observed in several healthy and tumor cells (Lan et al., 2005; Han et al., 2007). Dysfunctional calcium channel signaling has been observed in cognitive and cardiac decline, however, most of this research has so far been conducted in model organisms such as *Drosophila* (Lam et al., 2018; Navakkode et al., 2018). Within the atrio-ventricular region of rats sodium (Na_v_1.5) are downregulated with age whilst calcium (Ca_v_1.3) channels are upregulated, alongside augmented atrial-ventricular node functioning (Saeed et al., 2018). Similarly to this finding, an upregulation of L-type and osmotically activated calcium channels have been observed in human cardiomyopathy and aging, respectively (Jones et al., 2018; Sanchez-Alonso et al., 2018). An overview of ion channel dysfunctions and their relation to age-related disease is provided in Table 2. By controlling membrane potential and signal transduction pathways, ion channels on the surface of cells contribute to maintaining the proteostasis and homeostasis of systems. Therefore ion channels are incriminated in several age-related dysfunctions (Santulli and Marks, 2015; Rao et al., 2016). Because aging also involves physiological alterations of ion channel function, it is suggested that abnormal changes of ionic gradients can underlie age-dependent decline of physiological functions (Rao et al., 2016). With age, functional changes in ion channels lead to clinical phenotypes called channelopathies (Rao et al., 2016).

**Table 2:** Ionic channels and their age-related diseases by blocker/ligand matches (Guide to Immunopharmacology portal, http://www.guidetopharmacology.org/).

Ionic channel subtypes	Age related diseases	Blockers/ligands	Reference
Transient receptor potential channels (TRP1, TRPM3)	Polycystic kidney disease 2; polycystic kidney disease 2-like 1 protein;	No putative TRP1 blockers Cd^2+^, Ni^2+^ ligands	Oberwinkler et al., 2005
Voltage-gated potassium channels (Kv1.3)	Autoimmune diseases (i.e., diabetes, multiple sclerosis and rheumatoid arthritis)	Noxiustoxin;	Grissmer et al., 1994
		Charybdotoxin;	Attali et al., 1992
		Margatoxin	Garcia-Calvo et al., 1993
		Kaliotoxin	Rochat et al., 1998
		Maurotoxin
Voltage-gated calcium channels (Ca_v_1.4)	Ocular albinism, ocular albinism type 2	Dihydropyridine antagonists (verapamil, diltiazem)	Baumann et al., 2004
Calcium- and sodium-activated potassium channels (K_Ca_2.3)	Parkinson disease	K_Ca_2.3 blockers (Apamin, Leiurotoxin I)	Barfod et al., 2001
			Shakkottai et al., 2001
TRPV1 Transient receptor potential channels	Inflammatory bowel disease, Chron’s disease; Ulcerative colitis	Agatoxin	Barfod et al., 2001
TRPA1 Transient receptor potential channels	Inflammation, inflammatory pain and inflammatory diseases	Divalent cations modulators	Nagata et al., 2005
Cav2.2 Voltage-gated calcium channels	Renal and cardiovascular diseases	Omega-conotoxins	Lewis et al., 2000
Nav1.4 Voltage-gated sodium channels	Susceptibility to periods of hyperactivity	Saxitoxin	Penzotti et al., 2001
		Tetrodotoxin	Trimmer et al., 1989
		Mu-conotoxins	Safo et al., 2000
		Lidocaine	Makielski et al., 1999
Kv8.1 Voltage-gated potassium channels	Epileptic disease	K_v_8.1 is not functional on its own but modulates the properties of coexpressed K_v_2.1	Hugnot et al., 1996
Cav1.3 Voltage-gated calcium channels	Multiorgan disease	Cd^2+^; Verapamil dihydropyridine antagonist	Scholze et al., 2001
Kv4.3 Voltage gated potassium channels	Àtrial Fibrillation, Valvular heart disease	Phrixotoxin 1	Wang et al., 2000
K_Ca_2.1 Calcium- and sodium-activated potassium channels	Ataxia, epilepsy, memory disorders, pain and possibly schizophrenia and Parkinsons’s disease	NS8593 gating inhibitor	Strobaek et al., 2006
Nav1.6 Voltage-gated sodium channels	Motor end-plate disease	α scorpion toxins	Oliveira et al., 2004


### Cardiac Channelopathies

**FIGURE 1:**
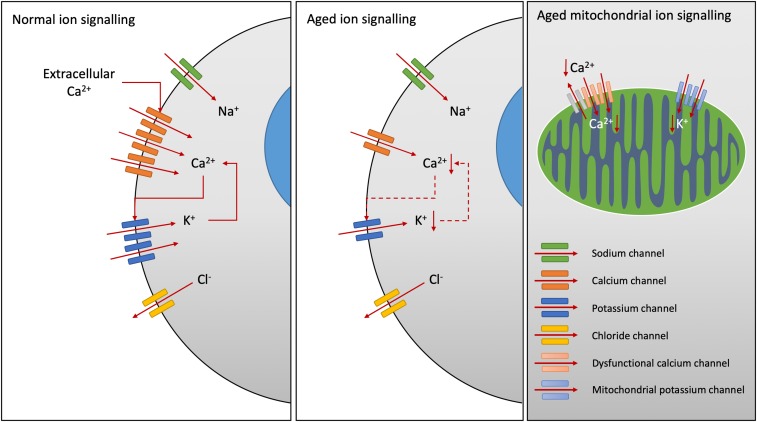
Age-related changes in ion channel function. Calcium (Ca^2+^) release-activated Ca^2+^ channels increase intracellular Ca^2+^ levels, activating K^+^ channel opening and sustained Ca^2+^ signaling, whilst efflux of chloride (Cl^-^) ions inhibits Ca^2+^ influx. Downregulation of Ca^2+^ channels has been demonstrated in Alzheimer’s disease. Decreased expression of Ca^2+^-activated K^+^ channels have been noted with aging, particularly within the smooth muscle cells of the vascular system reducing arterial tone. Within the mitochondria, reduced Ca^2+^ ion channel activity results in reduced Ca^2+^ cycling. Potassium channel expression on the mitochondria are also reduced with age in the heart sarcolemma.

The vascular system often shows decline in a number of age-related diseases. Atherosclerotic plaques have been observed in humans from early life but often do not have a clinical impact until later in life (Jones et al., 2017). Several evidence has shown that both endothelium and smooth muscle cells (SMCs) communicate through signal transduction. Within the numerous changes that may occur at vascular and/or cardiac levels, it is noteworthy that voltage-dependent and Ca^2+^-activated K^+^ (BK_Ca_) channels, abundant in vascular mural cell membranes, play an important role in vasodilatation and regulating coronary tone (Climent et al., 2017). In particular, they are activated by the vasodilator nitric oxide (NO). In age-related diseases, a reduction in the density of the BK_Ca_ alpha subunit in coronary smooth muscle was observed to be associated with NO release (Toro et al., 2002) meaning a decreased expression during aging (Figure 1). Despite endothelial dysfunction, changes in K^+^ channel expression and function are reported during aging in a young versus mature rat model (Simkin et al., 2015). Likewise, long-lasting L-type Ca^2+^ voltage dependent channels and high-conductance BK_Ca_ channels are recognized as key regulators of vascular and arterial tone through the NO-cyclic guanosine monophosphate (cGMP) pathway (Climent et al., 2017). Their activities are modulated by intracellular Ca^2+^ and their abundance diminished with aging.

Therefore, vascular dysfunction is one of the main factors linked to age-related diseases including cardiovascular and cerebrovascular diseases. It occurs through a progressive alteration of the structure and/or function of the vasculature. Within the signaling pathway, ion channels modulate ion fluxes by either activating K^+^ channels or inactivating Ca^2+^ channels leading to vasodilatation. In coronary smooth muscle during aging, a decrease in voltage-Ca^2+^-activated K^+^ channels has been described (Marijic et al., 2001) and an inhibitory effect of testosterone has been demonstrated (Scragg et al., 2004). Beside endothelium-dependent mechanisms, hormones (i.e., testosterone, oestrogen) are involved in vasorelaxation via ion channel modulation and activating several signaling pathways (i.e., Phosphoinostide 3-kinase (PI3K)/Akt-dependent pathways) in vascular endothelial cells (Hisamoto et al., 2001). Both hormone receptors and Ca^2+^ voltage-gated channels are on the plasma membranes suggesting some commonalities in subsequent signaling pathways (Núñez et al., 2018). With this respect, ion channels are nowadays the preferential molecular targets of future drug developments and ion channel modulators are promising medicines to reduce human pathophysiological changes (Testai et al., 2015; Bhattacharya and Biber, 2016; O’Conor et al., 2016).

### Ion Channels in the Aging Nervous System

Ion disturbance is present in neurological disorders associated with various types of voltage- and ligand-gated ion channel defects and/or mutations. For instance, implications of several voltage-gated sodium, potassium and calcium channel-subtype gene families are linked to dyskinesia, seizure, epilepsy, and ataxia pathogenesis (Simms et al., 2014). In neurons, changes in ionic fluxes occur very rapidly producing action potentials with fast depolarization, repolarization and signal propagation (Levitan and Kaczmarek, 2015). Neuronal signaling involves specific ion channels. The role of the ion channels in membrane physiology and brain homeostasis is essential, triggering nerve impulses and synaptic transmission. Many neurological disorders are caused due to altered function or mutation in ion channels (i.e., Alzheimer, Parkinson, Huntington, multiple sclerosis diseases) (Kumar et al., 2016). They are involved in propagation of action potential and secretion of neurotransmitters, therefore aberrant ion channels are considered of crucial negative influence in neurodegenerative disorders (Kumar et al., 2016). For instance, various calcium channels contribute to dysregulation of calcium homeostasis and play an important role in age-related changes (Navakkode et al., 2018). Recently, downregulation of the Ca_V_3.1 T-type calcium channel has been demonstrated in N2a cells and the 3xTg-AD mouse model of Alzheimer’s disease (Figure 1) (Rice et al., 2014).

Neurogenic inflammation and pain signaling are also mediated by specific members of the transient receptor potential (TRP) ion channel family. They are co-expressed in at least 25% of nociceptors (Bautista et al., 2006; Pingle et al., 2007). For example, TRPA1 mediates the inflammatory actions of environmental irritants and analgesic agents and enhance pain and inflammation (Matta et al., 2008). The same TRPA1 was shown to be activated in lung epithelial cells in response to cigarette smoke stress (Lin et al., 2015). This suggests some commonalities in the role of ion channels across various cell types and organs. Neuroinflammation and cognitive decline often appear concurrently in the aging brain, with inflammatory cytokines acting negatively on spatial memory (Blalock et al., 2003; Moore et al., 2009). The increased incidence of stroke with aging can bring about the production of inflammatory cytokines from microglia (Charolidi et al., 2015). Aging increases the production of interleukin (IL)-6 in the brain of aged mice, in a K_V_1.3-dependent manner. Alongside this, microglia in aged mice show increased expression of voltage-activated K^+^ channels, potentially enhancing IL-6 production and neuroinflammation with age (Schilling and Eder, 2015).

### Immuno-Channelopathies

In innate and adaptive immunity, regulation of membrane potential and calcium influx are determined by the equilibrium potentials of K^+^ (K_V_1.3, K_Ca_3.1), Na^+^ (TRPM4) and Cl^-^ channels in the plasma membrane (Feske et al., 2015). Following T cell activation, opening of Orai (the store-operated calcium channel) that encodes Ca^2+^ release–activated Ca^2+^ (CRAC) channels (also expressed in B cells, NK cells, macrophages, DCs, neutrophils) results in Ca^2+^ influx and subsequent opening of K_Ca_3.1 and K_V_1.3 channels (Figure 1) (Feske et al., 2015). The study of mechanisms underlying their function in lymphocytes, using ion channel inhibitors, revealed important roles of ionic signals in immune responses. For instance, the open state of K_Ca_3.1 and K_V_1.3 channels mediate K^+^ efflux and hyperpolarization of the plasma membrane, thereby sustaining Ca^2+^ influx (Figure 1). Alongside this, opening of Cl^-^ channels results in efflux of Cl^-^ ions that inhibits Ca^2+^ influx (Feske et al., 2015). Altered immune function through ion signaling can have profound effects on the development of age-related disease. Necrosis in the tumor microenvironment, which causes release of K^+^ ions into the extracellular space, has recently been shown to reduce effector T cell function in a TCR-dependent manner (Eil et al., 2016). Additionally, the activation of acid-sensing ion channels in microglia following stroke has been shown to increase the development of neuroinflammation in rats, tying ion signaling in immune cells to inflamm-aging (Yu et al., 2015).

## Ion Channels, Mitochondria and Aging

### Ion Channels Regulate Mitochondrial Functions

Mitochondria are often referred to as the powerhouse of the cell, however, their physiological role goes well beyond that (Chandel, 2015; Vakifahmetoglu-Norberg et al., 2017). Mitochondria are highly dynamic organelles regulating their structure in line with metabolism, redox signaling, mitochondrial deoxyribonucleic acid (DNA) maintenance and apoptosis (Vakifahmetoglu-Norberg et al., 2017). Besides from generating adenosine triphosphate (ATP) for cellular energy, mitochondria are also deeply involved in providing intermediates for cellular signaling and proliferation (Diebold and Chandel, 2016). Mitochondria can alter their size and organization as a result of mitochondrial fission and fusion in response to various intracellular and extracellular signals (Seo et al., 2010). Fission and fusion events occur to meet metabolic demands and for the removal of damaged/dysfunction mitochondria. The role of mitochondrial fission and fusion in facilitating metabolism has been researched extensively (Wai and Langer, 2016). Fused mitochondrial networks typically engage more oxidative pathways of metabolism, whilst fragmentation as a result of stress impairs the oxidative pathway and increases cellular demand on glycolysis (Wai and Langer, 2016).

Ion channels are intimately involved in regulating mitochondrial function (O’Rourke, 2007). The essential role of cationic hydrogen (H^+^) ion transfer in ATP production was noted as early as 1961 (Mitchell, 1961). H^+^ ions are pumped from the mitochondrial matrix into the intermembrane space by the flow of electrons through the electron transport chain. These ions are then utilized to drive the ATPase machinery and phosphorylate ATP, thus creating energy for the cell (Mitchell, 1961). The movement of ions across the mitochondrial membrane is also essential in establishing membrane potential and maintaining proton (H^+^) flux. Ions transported across the inner membrane include potassium (K^+^), sodium (Na^+^) and calcium (Ca^2+^), alongside H^+^ (O’Rourke, 2007). The most well-studied ion channel within the mitochondrion is the voltage-dependent anion channel, also known as VDAC, which is the primary route of metabolite and ion exchange across the outer mitochondrial membrane (Colombini, 2004).

Mitochondrial channelopathies have been found in aging, affecting the K^+^, Ca^2+^, VDAC and permeability transition pore (Ca^2+^; PTP) channels. Mitochondrial Ca^2+^ cycling is impaired with aging in neurons, resulting from reduced Ca^2+^ channel activity and reduced recovery after synaptosomal stimulation (Figure 1) (Satrustegui et al., 1996). This reduced calcium recovery rate results in reduced mitochondrial membrane potential and delayed repolarization, causing mitochondrial dysfunction with aging. This effect has been found in the heart of 2 year old senescent rats (Jahangir et al., 2001). In terms of potassium channels, it has been shown that their density on the surface of mitochondria significantly declines with age and with metabolic syndromes in the heart sarcolemma (Figure 1) (Ranki et al., 2002; Truong et al., 2016). This has been shown to reduce tolerance to ischemia-reperfusion and increased injury in aged guinea pig and rat hearts, and also humans (Roscoe et al., 2000; Kamada et al., 2008). These effects have repercussions in increasing susceptibility to myocardial infarction and reducing neuronal activity in the elderly as mitochondrial K^+^ channels have been shown to play a neuroprotective role in neurological reperfusion injury in postnatal mouse pups (Connors et al., 2015). Amyloid-β plaques in Alzheimer’s disease have been shown to increase intracellular calcium levels (Demuro et al., 2011). This increase in intracellular calcium, and uptake into the mitochondria through the VDAC and calcium uniporter, has been shown to increase mitochondrial stress responses and initiate apoptosis in rat cortical neurons *in vitro* and hippocampal slices *ex vivo* (Alberdi et al., 2010). Recent studies in Parkinson’s disease, have revealed that α-synuclein acts via the VDAC to promote mitochondrial toxicity of respiratory chain components in a yeast model of Parkinson’s (Rostovtseva et al., 2015).

### Mitochondrial Dysfunctions in Aging

In-born errors of metabolism and mitochondrial defects can have wide-spread effects on human physiology from birth (Vernon, 2015). These mitochondrial disorders are commonly characterized by symptoms such as vision loss, heart disease and dementia similar to that seen during aging, highlighting the crucial role of mitochondria in maintaining cellular and physiological function (Ganesh et al., 2017; Towbin and Jefferies, 2017; Sklirou and Lichter-Konecki, 2018). The onset of age-related pathologies have been linked to the development of mitochondrial dysfunction for some time, particularly in the development of Parkinson’s disease (Abou-Sleiman et al., 2006). Moreover, mitochondrial dysfunction has also been observed in cardiac disease, Alzheimer’s disease and more recently diabetic kidney disease (Lesnefsky et al., 2001; Reddy and Beal, 2008; Qi et al., 2017). Therefore, it is not hard to see the correlation between increasing mitochondrial dysfunction and the decline in physiological systems with aging. In fact, mitochondrial dysfunction was highlighted as one of the nine hallmarks of aging (López-Otín et al., 2013). Mitochondrial dysfunction manifests during normal aging, its aggravation accelerates aging and its amelioration in model organisms increases life span.

**FIGURE 2:**
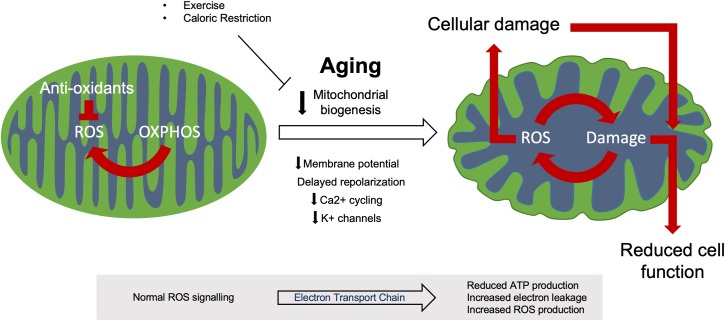
Mitochondrial dysfunction during aging. Healthy mitochondria produce ROS through regular oxidative (OXPHOS) activity which aid in normal cell processes, this ROS production is kept in check by various anti-oxidant systems to prevent oxidative damage. During aging, dysfunctional mitochondria accumulate due to reduced biogenesis and ROS control. This increased ROS production induces both further mitochondrial damage and cellular damage, resulting in reduced cell function and eventual apoptosis.

Mitochondrial biogenesis resulting from the growth and division of existing organelles maintains mitochondrial health and integrity, however, this process has been shown to be reduced with age in both animals and humans (Figure 2) (López-Lluch et al., 2008; Srivastava, 2017). The rate of loss of mitochondrial biogenesis with age is still argued, however, it has been shown to be augmented in response to physiological stimuli such as hormones and transcription factors. For example, oestrogen and progesterone promote whilst testosterone inhibits mitochondriogenesis in human brown adipose tissue *in vitro*, whilst the nuclear respiratory transcription factors (NRF1 and NRF2) influence the expression of mitochondrial respiratory genes (Rodriguez-Cuenca et al., 2007; Scarpulla et al., 2012).

Additionally, exercise and caloric restriction have been shown to enhance mitochondrial biogenesis whilst obesity and type 2 diabetes, which are prevalent in the aging population, have been shown to reduce it (Hebert et al., 2013; Heinonen et al., 2015). These mechanisms act directly by augmenting the expression of the mitochondrial biogenesis regulator peroxisome proliferator-activated receptor gamma coactivator 1-alpha (PGC-1α) and indirectly by mediating the activation of adenosine monophosphate-activated kinase (AMPK) which phosphorylates PGC-1α (Jager et al., 2007; Jornayvaz and Shulman, 2010). The PGC-1α transcriptional coactivator in line with NRF1 and NRF2 regulates the expression of nuclear encoded mitochondrial proteins (Lin et al., 2005). Hormone receptors including peroxisome proliferator-activated receptors alpha and gamma and oestrogen related receptor alpha also regulate the expression of mitochondrial genes in concert with PGC-1α (Lin et al., 2005). In response to stress AMPK induces mitophagy by inhibiting mammalian target of rapamycin (mTOR), whilst Sirtuin-1 phosphorylation by AMPK activates PGC-1α and stimulates mitochondrial biogenesis (Rodgers et al., 2005; Alers et al., 2012). In rats, reduced AMPK activity has been linked to reduced mitochondrial biogenesis and insulin resistance with age (Reznick et al., 2007; Qiang et al., 2007). Whilst caloric restriction has been shown to increase PGC-1α activity in primary rat hepatocytes (López-Lluch et al., 2006), the effect of caloric restriction on AMPK activity is under discussion with studies reporting both positive and negative results on AMPK signaling depending on the disease model studied (García-Prieto et al., 2015; Amaral et al., 2016; Bayliss et al., 2016).

During aging, the efficiency of mitochondrial electron transport chain weakens, thus reducing cellular ATP production and increasing electron leakage and reactive oxygen species (ROS) production in model organisms such as *Caenorhabditis elegans* and *Drosophila* (Figure 2) (Ferguson et al., 2005; Rea et al., 2007; Chistiakov et al., 2014). However, studies of human aging are conflicted over the relationship between electron transport chain activity and aging (Parker et al., 1989; Barrientos et al., 1996; Doria et al., 2012). Mitochondria have formed the basis of a number of theories examining the phenomenon of aging, the most well-studied and accepted being the free-radical theory of aging (Harman, 1992). This theory focuses on the increased production of ROS with age which causes progressive cellular damage (Figure 2). As ROS originate from the mitochondria, these powerful and essential organelles are the most susceptible to this oxidative damage (Belhadj Slimen et al., 2014). Mutations in mitochondrial DNA have previously been linked to impaired mitochondrial function and apoptosis in both murine and human brain and muscle tissue (Fayet et al., 2002; Kraytsberg et al., 2006; Li H. et al., 2017). This results in impaired tissue homeostasis, degeneration and the development of age-related phenotypes such as sarcopenia and neurodegeneration (Cha et al., 2015; Herbst et al., 2016). Whilst ROS have been linked to the increase in mitochondrial DNA mutations with aging, this phenomenon can occur in the absence of oxidative stress and has been attributed to replication errors (Seo et al., 2010; López-Otín et al., 2013). Additionally, the free radical theory of aging has recently been brought into question through studies in the model organism *C. elegans*. Here, more recent studies have shown that the absence of the antioxidant superoxide dismutase (SOD) does not affect lifespan unless the organism is put under stress (Van Raamsdonk and Hekimi, 2012). Loss of SOD2 specifically has been shown to increase lifespan in clk-1 ETC (Coenzyme Q) mutants whilst decreasing lifespan in isp-1 ETC (complex II) mutant worms (Van Raamsdonk and Hekimi, 2009). Additionally, mitochondrial ROS production is required for longevity in the *C. elegans* isp-1 and nuo-6 ETC mutants by protecting against mitochondrial dysfunction (Yee et al., 2014).

A pro-oxidative redox state has been implicated in the development of cardiovascular disease, inflammation, diabetes, neurodegeneration and cancer in human health (del Valle, 2011; Dai et al., 2014; Münzel et al., 2015). ROS have extensive effects within the cellular environment causing protein misfolding and aggregation, as well as oxidative damage to DNA (Nakamura and Lipton, 2017). Parkinson’s disease results from abnormalities in the expression of the protein Parkin, which aids in the autophagic degradation of dysfunctional and damaged mitochondria (Kazlauskaite and Muqit, 2015). Loss of this protein with age in humans and mice results in decreased autophagy of mitochondria (mitophagy), leading to the accumulation of damaged organelles, decreased function and neurodegeneration (Song et al., 2017; Zanon et al., 2017). A similar process has been observed in Alzheimer’s disease, where amyloid-β plaque accumulation leads to mitochondrial dysfunction and cellular toxicity. Studies in yeast have helped to delineate the mechanism behind this effect on mitochondrial function, whilst amyloid-β has also been shown to induce mitochondrial dysfunction in human neural stem cells (Mossmann et al., 2014; Chiang et al., 2016).

## Role of Mitochondria in the Regulation of Inflamm-Aging

### Inflammation and Age-Related Diseases

It is well established that there is an age-related dysregulation of pro/anti-inflammatory circulating cytokines which are increased threefold to fourfold in plasma or serum from elderly participants, termed inflamm-aging. Several studies have reported that this cytokine-related aging process appears to be related to a collection of age-related syndromes, including loss of muscle and bone mass, anemia, immune dysfunction and memory decline (Morley and Baumgartner, 2004; Reale, 2014). Westendorp has reported that persons with high levels of tumor necrosis factor alpha (TNFα) and low levels of IL-10 favor a protective role in infection and will have an extended lifespan, thus suggesting that the balance between pro- and anti-inflammatory cytokines influences longevity (Westendorp, 2004). However, the burden of disease has now shifted away from infectious diseases toward the chronic diseases that typically come with old age. In this regard IL-6 has been linked to the aging process and has been named the “geriatric cytokine” (Herpich et al., 2018). Muscle wastage and high mortality are associated with elevated serum levels of IL-6 in the elderly (Forcina et al., 2018; Ridker et al., 2018). For example, a one SD increase in IL-6 levels in elderly patients results in a 1.1–2.3 kg loss in grip strength, whilst acute injection of IL-6 in rats causes a 17% loss of myofibrillar protein (Visser et al., 2002; Haddad et al., 2005). This age-associated increase in production of IL-6 was recently proposed to be linked to the process of cellular senescence. Senescent cells, which number increases with aging, were shown to have a pro-inflammatory secretory profile, including IL-6, which is part of the senescence associated secretory phenotype (SASP) (Watanabe et al., 2017). Alongside IL-6 the concentration of TNFα, a pro-inflammatory cytokine produced by macrophages and adipocytes, is significantly increased in centenarians compared with younger subjects (Bruunsgaard et al., 1999; Holmes et al., 2009). This increase was associated with a greater incidence of both Alzheimer’s disease and generalized atherosclerosis in centenarians (Bruunsgaard et al., 1999; Holmes et al., 2009). This chronic, sterile, low-grade inflammation called inflamm-aging contributes to the pathogenesis of age-related diseases such as those summarized in Table 3. SASP cytokines such as IL-6 and TNFα are regulated by molecules such as IL-10 (Stenvinkel et al., 2005). The interaction between pro- and anti-inflammatory molecules is complex and in the context of cardiovascular disease strongly suggests that dysregulation of this interplay may lead to complications such as end stage renal disease.

**Table 3:** Cytokines and their age-related-diseases.

Age-related-diseases	Principal associated	cytokine	Reference
Sepsis	↑ IL-6 level	→ ↑ risk	Starr and Saito, 2014
Alzheimer disease	↑IL-6 Level	→ ↑ risk	Banks and Morley, 2003
	↑IL-1β level		Wilson et al., 2002
			Licastro et al., 2000
	↓G-CSF	→ ↑ risk	Barber et al., 2012
Cachexia syndrome	↑TNFα level	→ ↑ risk	Baez-Franceschi and Morley, 1999
Dilated cardiomyopathy	↑TNFα level	→ ↑ risk	Levine et al., 1990
Atherosclerosis and osteopenia	↑IL-6 level	→ ↑ risk	Hak et al., 2000
	↑TNFα level		Jørgensen et al., 2001
	↑ IL-1β level		
Cognitive decline	↑IL-6 level	→ ↑ risk	Engelhart et al., 2004
	↑TNFα level		Schram et al., 2007
			Weaver et al., 2002
Acute stroke	↑IL-6 level	→ ↑ risk	Jung et al., 2010
	↑TNFα level		Stankowski and Gupta, 2011
	↑ IL-10 level	→↓ risk	Van Exel et al., 2002
	↑ TGF-β level		Pang et al., 2001


Recent advances have shown that the pro-inflammatory capacity of cells is regulated by the inflammasome (Guo et al., 2015). The inflammasome is a multiprotein intracellular complex that forms in response to stress (pathogenic microorganisms/sterile stressors) and results in the release of the pro-inflammatory cytokines IL-1β and IL-18 (Guo et al., 2015). IL-1β induces IL-2 and TNFα through the activation of T-helper cells, and produces tissue inflammatory actions by activating cyclooxygenase-2 to produce prostaglandin E2, inducible intercellular adhesion molecules and NO (Raeburn et al., 2002). IL-1β causes fever, anorexia, sickness behavior and a decline in the ability to acquire and retain memory in mice (Banks et al., 2001). It was also reported that IL-1β may increase the production of amyloidal precursor protein within the central nervous system and the development of neurodegenerative disorders such as Alzheimer’s disease, with increased plasma levels of pro-inflammatory cytokines IL-1 and IL-6 also observed in these patients (Licastro et al., 2000; Wilson et al., 2002; Banks and Morley, 2003; Shaftel et al., 2008).

Age-related inflammation has recently been postulated as a concomitant adaptation to the metabolic shifts observed during aging (Franceschi et al., 2018). This metabolic shift toward decreased mitochondrial respiration and increased glycolysis is observed within the model organism *C. elegans* (Feng et al., 2016), as well as in rat hepatocytes and human skeletal muscle (Hagen et al., 1997; Gouspillou et al., 2014). In aging bone, impaired oxidative metabolism leading to a glycolytic shift has been identified in mice due to PTP opening and mitochondrial dysfunction (Shum et al., 2016). However, human fibroblasts from aged individuals display a decrease in glycolytic flux and lactate output and increase in oxygen consumption rate and ATP levels (Son et al., 2017), suggesting this age-related metabolic shift may be tissue specific. This metabolic adaptation and concurrent inflammation with age is thought to result in chronic inflammation driven by nutrient excess, obesity and regulated by gut microbiota (Franceschi et al., 2018). This is supported by the fact TNFα promotes anorexia, stimulates lipolysis and inhibits lipoprotein lipase, leading to cachexia syndrome in older persons. This implicates cytokines in feeding behavior and their effects in the elderly (Baez-Franceschi and Morley, 1999). This meta-inflammation hypothesis supports the potential role of mitochondria, an essential keychain of the metabolic regulations, in driving age-related inflammation. As discussed above (i) mitochondria are involved in immune-metabolic adaptation (ii) mitochondrial dysregulation is a core hallmark of aging and senescence (iii) mitochondrial functions rely highly on ion channel biology. Altogether this suggests an unexplored role of ion channels in the regulation of inflammation.

### Ion Channels, Mitochondria and SASP

#### Immune Cells

It has been more than 30 decades since expression of ion channels in non-excitable immune cells (i.e., T lymphocytes) was first described (DeCoursey et al., 1985). Besides their role in cell excitability, K_V_ channels are also involved in regulating cellular secretion and in the differentiation and growth of non-excitable cells. In particular, K_V_ channels have been implicated in the proliferation of many cell types and are usually down- or up-regulated in immune cells of patients with cancerous diseases (Pardo, 2004; Pardo et al., 2005). On the surface of immune cells, a variety of ion channel subtypes are displayed. K_V_1.3 are the most well-described potassium channel subtype involved in the inflammation process and their dysfunction is associated to altered T function, through impacting the calcium influx and contributing to age-related changes of T cell function (Kollár et al., 2015).

The most recent advances in the field of senescence, aging and inflammation relate to the concept of the SASP. SASP refers to the increased secretion of inflammatory cytokines, chemokines and growth factors secreted by senescent cells (Watanabe et al., 2017). The most common group of secreted SASP factors are the major pro-inflammatory cytokines IL-1β, IL-6 and IL-8, produced via augmented NF-κB and mTOR signaling in senescent human fibroblasts and embryonic kidney cells (Chien et al., 2011; Herranz et al., 2015). A causal relationship between systemic inflammation and the prevalence of generalized age-related osteoporosis has been observed in the elderly (Yun and Lee, 2004; Ginaldi et al., 2005). Inflammatory cytokines such as IL-6, TNF-α and IL-1β produced through SASP (Watanabe et al., 2017), are stimulators of osteoclast activity and are linked to the development of atherosclerosis and osteopenia (Hak et al., 2000; Jørgensen et al., 2001). SASP factors have simultaneously been shown to affect injury recovery. Low levels of IL-6 were significantly correlated with better functional and muscular recovery after femoral neck fracture in women of good health aged 65 and over (Miller et al., 2006, 2008).

**FIGURE 3:**
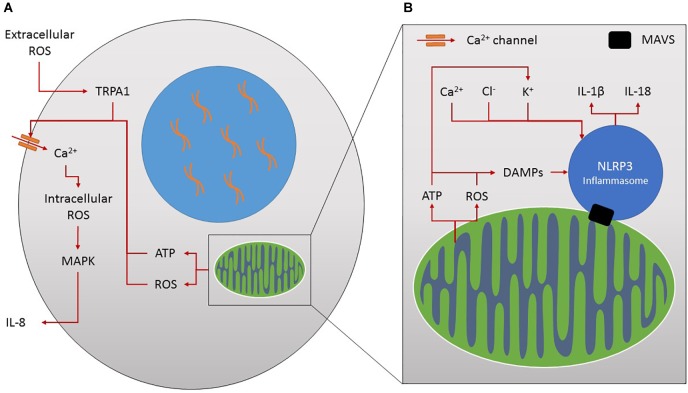
**(A)** Ion channels and inflammation. Increased extracellular reactive oxygen species (ROS) stimulates TRPA1 and calcium (Ca^2+^) influx. This increases intracellular ROS production, MAPK activation and pro-inflammatory IL-8 release. Mitochondria produce ROS and ATP through their normal activities which affect ion flux, for example by increasing calcium influx into the cell by increasing calcium ion channel activity and stimulating further ROS production. In turn the secretion of inflammatory molecules such as IL-8 is stimulated. **(B)** Mitochondria, ions and inflammation. Mitochondria form the major platform for NLRP3 inflammasome assembly through the mitochondrial antiviral signalling protein (MAVS). Mitochondria activate this inflammasome complex by releasing damage associated molecular patterns (DAMPs) such as ROS and ATP leading to the maturation of IL-1β and IL-18 inflammatory molecules. Similarly, increased influx of ions such as calcium, chloride and potassium can also influence inflammasome activation.

Activation of inflammasomes, particularly the nod-like receptor P3 (NLRP3) inflammasome, is highly dependent on the activity and integration of the mitochondria. The mitochondrial antiviral signaling protein (MAVS) adaptor is required for recruitment of NLRP3 to the mitochondria and its activation (Figure 3B), whilst the endoplasmic reticulum-mitochondrial junction is also integral to inflammasome activation (Raturi and Simmen, 2013; Subramanian et al., 2013). Dysfunctional mitochondria have been linked to inflammation for some time, and have also been implicated in the inflamm-aging process (Picca et al., 2017). Damage-associated molecular patterns (DAMPs) accumulate with age as a result of the release of ROS and ATP from damaged mitochondria (Kapetanovic et al., 2015). Although mitochondria have been present in animal cells for millions of years, it is believed that mitochondria originated from bacteria and can be seen by the body as pathogen-associated molecular patterns (PAMPs) (Krysko et al., 2011). Both DAMPs and PAMPs induce an immune response by triggering the activation of inflammasomes, including the NLRP3 inflammasome in innate immune cells such as macrophages, driving maturation of IL-1β and IL-18 pro-inflammatory cytokines (Figure 3B) (Kapetanovic et al., 2015).

It is also tempting to speculate on the role of ion channels in the activation of the inflammasome. Potassium and chlorine efflux, alongside calcium influx, have been shown to influence NLRP3 inflammasome activation (Figure 3B) (Jo et al., 2016; Hafner-Bratkovic and Pelegrin, 2018). ATP, as a common activator of NLRP3, induces decreased levels of potassium and alters other ionic contents within the cell (Jo et al., 2016), essential for inflammasome activation in monocytes/macrophages (Petrilli et al., 2007). In fact the intracellular chloride channel has been shown to act downstream of potassium efflux, as a result of mitochondrial ROS generation, to promote NLRP3 activation in murine macrophages and a human monocytic cell line (Tang et al., 2017). Inflammasome activation itself has also been shown to affect potassium, chlorine and calcium homeostasis contributing to cytokine release in cells of the innate immune system (Perregaux and Gabel, 1994; Hafner-Bratkovic and Pelegrin, 2018). Chronic inflammation, Alzheimer’s disease and metabolic diseases such as Type 2 diabetes have been linked to aberrant activation of the NLRP3 inflammasome by damaged mitochondria and dysregulated ion flux (Jo et al., 2016). Therapeutic interventions to modulate the adverse and overlapping effects of the numerous different inflammatory mediators on each ion transport system could target adversely affected ion transport systems directly and locally.

#### Other Cells

The expression or function of most ion channels can be modulated by cytokines, prostaglandins, leukotrienes and ROS resulting from inflammation. Key pathways in this interaction are cyclic nucleotide, phosphoinositide and mitogen-activated protein kinase (MAPK)-mediated signaling, direct modification by ROS-like NO, ATP or protons and disruption of the cytoskeleton in neuronal and epithelial cells (Eisenhut and Wallace, 2011). The increased levels of extracellular ROS following stress, such as smoke exposure, leads to a 2.5-fold increase in Ca^2+^ influx following TRPA1 activation in human bronchial epithelial cells (Lin et al., 2015). This contributes to increased intracellular ROS (via NADPH oxidase) activating the MAPK pathway and increasing the secretion of IL-8 in innate immune cells (Figure 3A) (Lin et al., 2015). The relationship between altered chloride channels and airway-related inflammation was recently reviewed (Sala-Rabanal et al., 2015) and also included the chloride channel regulator, the calcium-activated chloride channel (TMEM16A), and chloride exchangers (SLC26A4 and SLC26A9), as potential regulators of inflammation.

Additionally, ion channels have been shown to be involved in the development of senescence. For example, expression of the sodium voltage-gated channel SCN9A maintains cellular senescence induced by oncogene expression, whereas loss of this channel allows mammary epithelial cells to escape senescence development (Warnier et al., 2018). SCN9A is upregulated in an NF-κB-dependent manner following oncogene induced senescence, and induces plasma membrane depolarisation in a manner similar to that of calcium and potassium ion channels (Wiel et al., 2014; Warnier et al., 2018). TRP channel, TRPC5, has also been implicated in the development of senescence in mouse vascular endothelial cells in a ROS-dependent manner, with β-galactosidase staining reduced in cells lacking this gene (Li Z. et al., 2017). Therefore, therapeutics targeting ion channels could be useful in restoring endothelial cell function and reducing the incidence of cardiac disease in this context (Minamino et al., 2002). In contrast to the reversal of senescence, small molecule activation of K_V_11.3 plasma-membrane potassium channel have shown promise in inducing p16^INK4A^-dependent senescence in melanoma cells *in vivo*, where the induction of senescence is preferable (Perez-Neut et al., 2016). Induction of senescence involved an AMPK-dependent cellular stress response following a rapid increase in intracellular calcium as evidenced by increased rates of autophagy. Consequently, therapeutics designed to activate or reverse senescence could both be of use in the aging population.

## Intervention Strategies to Restore Mitochondrial Function

The removal of senescent cells is a strategy used to improve physical capacity in old age. The use of senolytics to clear senescent cells by exploiting their apoptotic pathways have already shown promise in pre-clinical studies alleviating the burden of SASP (Kirkland and Tchkonia, 2017). Particularly the SASP components IL-6, IL-1α and TGF-β show decreased expression with the use of senolytics in senescent murine hematopoietic stem cells, human lung fibroblasts and a murine model of pulmonary fibrosis (Chang et al., 2016; Schafer et al., 2017). Consequently, the removal of senescent cells should reduce the overall pro-inflammatory profile in older individuals. In senescent human fibroblasts the anti-aging drug resveratrol, has recently been shown to reduce SASP potentially through targeting mitochondrial dysfunction in a p16^INK4A^-dependent mechanism (Pitozzi et al., 2013; Kirkland and Tchkonia, 2017). The senolytic properties of resveratrol in senescent porcine aortic endothelial cells involve mitochondrial Ca^2+^ overload-induced apoptosis (Madreiter-Sokolowski et al., 2019). However, the difficulty resides in the specific targeting of senescent cells in humans, as expression of senescent hallmarks is highly variable in a tissue-specific manner, as well as in the potential detrimental effect of this removal (Hudgins et al., 2018). Considering aging as an adaptation, it can be difficult to conceive that removal of senescent cells will only have beneficial effects. This may vary from organ to organ and as of today it is difficult to predict the tissue-specific effects of senolytics without further testing. Other intervention strategies should now be considered.

Many interventions affecting mitochondrial function have been shown to affect lifespan and healthspan. Inducing mild mitochondrial stress throughout the life course may result in better outcomes later in life due to a programming effect, termed mitohormesis (Yun and Finkel, 2014). As the ROS theory of aging is so popular, anti-oxidants were one of the first drugs utilized to ameliorate mitochondrial dysfunction during aging (Cutler, 1984; Aversa et al., 2016). Whilst ROS have long since been believed to cause cellular dysfunction through the oxidative damage they cause, their role within the cell is much more complex. The generation of mitochondrial-targeted anti-oxidants have allowed the effects of anti-oxidants specifically on mitochondria and metabolism to be studied more effectively. These antioxidants have been shown to protect the mitochondria from further oxidative damage more effectively than natural anti-oxidants such as Vitamin E in rat liver mitochondria and human osteosarcoma cells, sustaining their function during oxidative stress (Smith et al., 1999). Whilst anti-oxidant supplementation can reduce oxidative stress, they can also have detrimental effects on health in the long term (Peternelj and Coombes, 2011; Desjardins et al., 2017). This is because ROS are essential for optimal cellular signaling, including that of ion channels. Anti-oxidants may not be beneficial as an intervention for restoring mitochondrial function as they may over-compensate, detrimentally affecting vital cellular functions and organism physiology. For example, anti-oxidants have been shown to affect ion channel activity and can block ATP-sensitive potassium channels in feline cerebral arterioles, inhibiting the action of hydrogen peroxide in vasodilation (Wei Enoch et al., 1998).

Metformin, used for the treatment of diabetes, has wide-ranging effects on mitochondrial function through multiple mechanisms (Viollet et al., 2012). These mechanisms include decreased gluconeogenesis and mitochondrial complex I inhibition resulting in partially inhibited metabolism (Viollet et al., 2012). This response increases the activation of AMPK as a response to metabolic stress. AMPK as the master regulator of metabolism has the ability to induce many beneficial changes within the mitochondria, from inducing mitochondrial biogenesis to employing autophagy to regain energy homeostasis (Hardie et al., 2012). This allows healthy mitochondria to proliferate, whilst damaged mitochondria are removed through autophagy and broken down. AMPK also engages oxidative phosphorylation which has been shown to promote longevity in some organisms (Hardie et al., 2012).

This may account for the recent call to repurpose drugs such as metformin for slowing down the appearance of age-associated diseases (by slowing the aging process itself). Contrary to recent data implicating AMPK-activation in promoting longevity, activation of AMPK has been shown to induce T cell senescence (Lanna et al., 2014), resulting in defective TCR signaling and reduced proliferation. Similarly, NK cell function has been found to be reduced in an AMPK-dependent manner in individuals aged 70 and over (Müller-Durovic et al., 2016). This may hinder the use of metformin as a potential healthspan extending drug in clinical trials. In terms of ion channels, polyspecific cation transporters such as OCT1 (SLC22A1) are required for accumulation of intracellular metformin and its interaction with mitochondria and endoplasmic reticulum (Chien et al., 2015). This interaction has been observed in human embryonic kidney cells and mouse liver, where OCT1 deletion results in a similar action on metabolism as metformin by disrupting glycolysis and activating AMPK (Chen et al., 2014). Metformin has been shown to directly interact with the intracellular chloride channel (CLIC1) in some human cells (Gritti et al., 2014). In human glioblastoma stem cells inactivation of CLIC1 by metformin inhibits the chloride current and induces cell cycle arrest (Gritti et al., 2014). Metformin has been shown to have no effect on Ca^2+^ current amplitude and K^+^-contraction in smooth arterial muscle cells and therefore does not affect vasodilation or contraction in guinea-pig arterial SMCs (Nakamura et al., 1998). In adult rat myocytes, however, metformin has been shown to normalize aberrant intracellular Ca^2+^ clearing induced by high glucose (Ren et al., 1999). This finding may implicate metformin in increasing cardioprotection in diabetic patients.

Long-term caloric restriction has previously been shown to reduce oxidative mitochondrial DNA damage with age in rat liver and also decrease ROS production rate within the mitochondria (Lopez-Torres et al., 2002). Therefore, utilizing caloric restriction as a therapeutic strategy to combat aging has received wide-spread attention. Caloric restriction has long been shown to extend lifespan in a number of animal models ranging from monkeys to yeast, however, studying the effects in humans has been challenging due to ethical and methodological concerns (Heilbronn and Ravussin, 2003). From studies in rhesus and cynomolgus monkeys it has been shown that caloric restriction can extend healthspan by delaying the onset of age-related chronic diseases, such as cardiovascular disease and insulin insensitivity (Type II Diabetes) (Cefalu et al., 2004; Mattison et al., 2017). Cefalu and colleagues reported a twofold increased risk for age-related morbidities in control animals, extending from frailty through to diabetes and cancer compared to those on a calorie restricted diets in an examination of two different studies (Cefalu et al., 2004). Utilizing very low-calorie diets as a short-term alternative for studying the effects of caloric restriction, has shown that reducing caloric intake in humans brings about a series of beneficial health effects. The most striking finding is that an 8-week low-calorie diet can achieve remission of impaired insulin responses, which can be sustained for at least 6 months (Steven et al., 2016). Similar studies have also shown to reduce inflammatory and oxidative stress biomarkers (Merra et al., 2017) and improve cardiovascular health in humans (Wei et al., 2017). Although only short-term, these studies show that diets utilizing caloric restriction can benefit health and may represent a strategy for delaying age-related disease progression which can be used as both an intervention and a preventative strategy. Caloric restriction has been shown to activate AMPK-mTOR signaling inducing mitochondrial biogenesis, improved energy homeostasis and increased lifespan (Dong et al., 2017; Marin et al., 2017; Wierman et al., 2017). Additionally, caloric restriction has been shown to increase Ca^2+^ retention and buffering capacity in mouse liver and rat brain mitochondria, resulting in reduced ischaemia-reperfusion damage and reduced excitotoxicity (Amigo et al., 2017; Menezes-Filho et al., 2017).

The positive effects of exercise on increasing mitochondrial health and increasing healthspan have been known for some time. The global effects of physical exercise on the hallmarks of aging has been reviewed recently (Rebelo-Marques et al., 2018). Exercise training in male young and old mice has been shown to promote biogenesis of mitochondria and mitochondrial autophagy and suppress pro-inflammatory cytokine production by up to 49% (Zhang et al., 2018). A similar effect has also been in seen in humans, whereby a significant increase in anti-oxidant levels and activity were observed in old active but not old sedentary individuals after incremental exercise (Bouzid et al., 2018). In aged mice an augmented oxidative profile following a low-impact and accessible swimming regime has been shown to aid in recovery after myocardial infarction even at durations as short as 15 min, by decreasing ROS production by 48% (Zhao et al., 2018). In murine studies, the beneficial effects of exercise on mitochondrial health appear to be dependent on the induction of PGC-1α expression, however, this link has yet to be confirmed within human skeletal muscle (Halling et al., 2017). Two months of training in 70-year old individuals has revealed that mitochondrial Ca^2+^ uptake can also be improved by exercise by increasing calcium uniporter expression (Zampieri et al., 2016). Exercise intervention in the elderly has come under scrutiny in the past due to the limited effects on mortality (West and Jones, 2013), however, a more recent meta-analysis looking at exercise prescription has confirmed reduced cardiovascular mortality from 10.4 to 7.6% (Anderson et al., 2016). Additionally, exercise has been shown to have positive effects on cognitive aging (Conner et al., 2017). As the elderly often suffer from multiple diseases taking additional medication and altering diet is often unfavorable. In this respect exercise is different from the other interventions described in this section and can also have advantageous social benefits. In conclusion this suggests that regular exercise in elderly can have advantageous effects on healthy ageing, decreasing both the hallmarks of aging and development of age-related disease.

## Perspectives

Mitochondria are an essential entity of cells. Ion channels are an essential regulator of mitochondrial functions. Knowing the role of mitochondria in cellular energy building and the consequences of its dysregulation, it becomes evident that a deeper understanding of ion channel biology should enable to provide better supportive strategies in the context of aging. The family of ion channels described in this review are involved in several physiological systems and influence immune responses, neuronal signaling and cardiovascular functions which are all very essential systems. In view of extending healthspan one should then consider investigating at potential ion channel modulators to influence mitochondria in an aging organism. Indeed, ion channels are currently being validated as targets for therapeutic development and their precise role during regulated and unregulated cell death is being investigated (Kunzelmann, 2016; Douthwaite et al., 2017; Grandi and Dobrev, 2017). Recent meta-analyses have concluded that the use of calcium-channel blockers can reduce the risk of developing Parkinson’s disease by up to 30% (Lang et al., 2015), and blockers of Ca_V_1.3 for treating Parkinson’s by reducing mitochondrial impairment and neuroinflammation are currently in Phase III clinical trials (Swart and Hurley, 2016). While the clinical effects of such interventions may be observed in a long-term manner, the investigation of oxidative stress and inflamm-aging should enable to have closer outcomes to look at. Indeed, chronic inflammation has been associated with a myriad of diseases and aged individuals with the low inflammation were shown to be distant from the risk category for most age-related diseases.

## Author Contributions

AL conceived and coordinated the writing of the manuscript. MS, BY-L, BB-Z, SP, and AL contributed to writing of this manuscript. MS and AL prepared the figures.

## Conflict of Interest Statement

The authors declare that the research was conducted in the absence of any commercial or financial relationships that could be construed as a potential conflict of interest.
